# Chelation Motifs Affecting Metal-dependent Viral Enzymes: *N′*-acylhydrazone Ligands as Dual Target Inhibitors of HIV-1 Integrase and Reverse Transcriptase Ribonuclease H Domain

**DOI:** 10.3389/fmicb.2017.00440

**Published:** 2017-03-20

**Authors:** Mauro Carcelli, Dominga Rogolino, Anna Gatti, Nicolino Pala, Angela Corona, Alessia Caredda, Enzo Tramontano, Christophe Pannecouque, Lieve Naesens, Francesca Esposito

**Affiliations:** ^1^Department of Chemistry, University of ParmaParma, Italy; ^2^Research Interuniversity Consortium Chemistry of Metals in Biological Systems Parma Unit, University of ParmaParma, Italy; ^3^Department of Chemistry and Pharmacy, University of SassariSassari, Italy; ^4^Department of Life and Environmental Sciences, University of Cagliari, Cittadella Universitaria di MonserratoCagliari, Italy; ^5^Genetics and Biomedical Research institute, National Research CouncilMonserrato, Italy; ^6^Rega Institute for Medical Research, KU LeuvenLeuven, Belgium

**Keywords:** antiviral agents, HIV-1 integrase, RNase H, chelating pharmacophore, dual inhibitors, acylhydrazone

## Abstract

Human immunodeficiency virus type 1 (HIV-1) infection, still represent a serious global health emergency. The chronic toxicity derived from the current anti-retroviral therapy limits the prolonged use of several antiretroviral agents, continuously requiring the discovery of new antiviral agents with innovative strategies of action. In particular, the development of single molecules targeting two proteins (dual inhibitors) is one of the current main goals in drug discovery. In this contest, metal-chelating molecules have been extensively explored as potential inhibitors of viral metal-dependent enzymes, resulting in some important classes of antiviral agents. Inhibition of HIV Integrase (IN) is, in this sense, paradigmatic. HIV-1 IN and Reverse Transcriptase-associated Ribonuclease H (RNase H) active sites show structural homologies, with the presence of two Mg(II) cofactors, hence it seems possible to inhibit both enzymes by means of chelating ligands with analogous structural features. Here we present a series of *N′*-acylhydrazone ligands with groups able to chelate the Mg(II) hard Lewis acid ions in the active sites of both the enzymes, resulting in dual inhibitors with micromolar and even nanomolar activities. The most interesting identified *N′*-acylhydrazone analog, compound **18**, shows dual RNase H-IN inhibition and it is also able to inhibit viral replication in cell-based antiviral assays in the low micromolar range. Computational modeling studies were also conducted to explore the binding attitudes of some model ligands within the active site of both the enzymes.

## Introduction

Human Immunodeficiency Virus type 1 (HIV-1) is responsible of the infection of 30 million people worldwide. The ssRNA HIV-1 genome retrotranscription into proviral dsDNA is a fundamental step in the HIV-1 replication cycle. This process is carried out by the viral coded RT, a multifunctional protein that catalyzes different reactions combining the RNA- and DNA-dependent DNA polymerase and RNase H activities, both essential for viral replication (Esposito and Tramontano, 2014). After the retrotranscription process, the viral enzyme IN allows the integration of the HIV-1 genome into the host cell chromosome, through two essential catalytic reactions, named 3′-processing and strand-transfer (Esposito et al., 2015). Despite an effective antiretroviral therapy has been developed, the incidence of HIV infection continues to raise. The development of new drugs with new mode of actions, such as single molecules that may act on two different target enzymes or two different catalytic functions (Patyar et al., 2011), would reduce the number of administered drugs, their chronic toxicity and the chance of selecting drug resistant viruses (Costi et al., 2014; Esposito and Tramontano, 2014); hence it remains one of the current main goals in drug discovery.

Metal-chelating molecules have been extensively studied as antivirals, resulting in the individuation of important classes of metal-dependent enzyme inhibitors (Ronconi and Sadler, 2007; Hocharoen and Cowan, 2009; Liao et al., 2010; Rogolino et al., 2012; Barry and Sadler, 2013). In particular, drugs targeting HIV-1 IN have evolved into an important component of the currently used clinical protocols. Their mechanism of action involves the chelation of the magnesium cofactors within the active site of the HIV-1 IN enzyme. The first clinically developed IN inhibitor (INI), raltegravir (Isentress^®^), was approved in late 2007 (Summa et al., 2008; Hicks and Gulick, 2009). In 2012, elvitegravir (Sato et al., 2006) and in 2013 dolutegravir (Kawasuji et al., 2013) jointed the therapeutic pool, and other chelating IN inhibitors are currently in clinical trials (Karmon and Markowitz, 2013).

**FIGURE 1:**
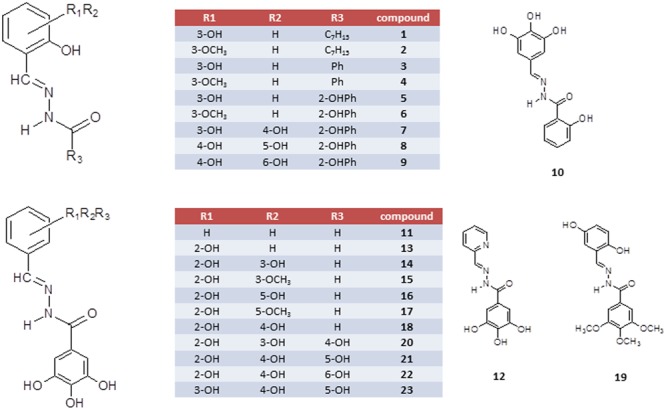
**Schematic representation of *N′*-acylhydrazones 1–23**.

Since HIV IN catalytic domain exhibits striking structural similarities with the viral RT-associated RNase H domain (Rogolino et al., 2012), the development of dual-acting drugs targeting both viral IN and HIV-1 RT-associated RNase H function has been proposed as an interesting strategy (Didierjean et al., 2005; Billamboz et al., 2008; Marchand et al., 2008; Corona et al., 2014a; Costi et al., 2014; Cuzzucoli Crucitti et al., 2015). In fact, both HIV-1 IN and RNase H domains belong to the polynucleotidyl transferases superfamily (Esposito and Tramontano, 2014); these similarities allow to investigate the structural features that a compound should hold to achieve a dual IN/RNase H inhibition, taking advantage of the interactions that an inhibitory compound can make with the amino-acidic lateral chains that surround the catalytic core of both IN and RNase H (Corona et al., 2016). It is worth noting that, despite the RNase H function is a promising target for drug development (Corona et al., 2014b) and different classes of compounds were reported as potent inhibitors of the HIV-1 RNase H function (Esposito et al., 2012; Corona et al., 2013), no RNase H inhibitors have moved toward an advanced stage of (pre)clinical development. *N′*-acylhydrazones represent an interesting class of chelating ligands with a broad spectrum of antiviral activities (Borkow et al., 1997; Rollas and Küçükgüzel, 2007), including HIV (Gong et al., 2011; Sanchez et al., 2013; Herschhorn et al., 2014), vaccinia virus (Abdel-Aal et al., 2006) and influenza virus (Chen et al., 2014). Recently, we focused our attention on a family of *N′*-acylhydrazones which proved to be versatile chelating inhibitors of the influenza virus PA endonuclease (Carcelli et al., 2016). We also identified some *N′*-acylhydrazone metal complexes with promising *in vitro* antiviral activity against various DNA- and RNA-viruses (Rogolino et al., 2015). Based on the functional similarity between HIV IN, HIV RNase H and the influenza PA endonuclease (Rogolino et al., 2012), we decided to evaluate a panel of *N′*-acylhydrazones against both HIV IN and RNase H, with the aim of identifying a unique chelating motif effective across metal-dependent enzymes of diverse viruses. Herein we present the biological evaluation in both enzymatic and cellular assays of a series of chelating *N′*-acylhydrazones (compounds **1–23, Figure 1**).

The structure-activity relationship for these compounds was analyzed, based on their presumed metal binding modes, thus identifying the chelating features involved in dual inhibition of both HIV IN and RNase H. Interestingly, some of these compounds (i.e., **18, 20, 21** and **23**) inhibit the RT-associated RNase H function in the micromolar and high nanomolar range. In particular, compound **18** is also able to inhibit viral replication in cell-based antiviral assays in the low micromolar range, representing one of the best inhibition profiles so far reported. Furthermore, computational modeling studies were conducted for compound **18** and some model ligands to explore the binding mode of these compounds within the active sites of the two HIV enzymes.

## Materials and Methods

All reagents of commercial quality were used without further purification. The purity of the compounds was determined by elemental analysis and verified to be ≥95%. NMR spectra were recorded at 25°C on a Bruker Avance 400 FT spectrophotometer. The attenuated total reflectance IR spectra were recorded by means of a Nicolet-Nexus (Thermo Fisher) spectrophotometer by using a diamond crystal plate in the range of 4000–400 cm^-1^. Elemental analyses were performed by using a FlashEA 1112 series CHNS/O analyzer (Thermo Fisher) with gas-chromatographic separation. Electrospray mass spectral analyses (ESI-MS) were performed with an electrospray ionization (ESI) time-of-flight Micromass 4LCZ spectrometer. MS spectra were acquired in positive EI mode by means of a direct exposure probe mounting on the tip of a Re-filament with a DSQII Thermo Fisher apparatus, equipped with a single quadrupole analyzer.

### Chemistry

Compounds **1–23** were synthesized following literature methods (Congiu and Onnis, 2013; Carcelli et al., 2016; Kim et al., 2016). Their characterization is reported in the Supplementary Materials.

### HIV-1 RT-associated RNase H Assays

Human immunodeficiency virus type 1 heterodimeric RT was expressed and purified as previously described (Esposito et al., 2011; Meleddu et al., 2014). The HIV-1 RT-associated RNase H activity was measured as described (Corona et al., 2014a; Meleddu et al., 2015). Briefly, 20 ng of HIV-1 RT was incubated in 100 μL reaction volume containing 50 mM Tris-HCl (pH 7.8), 6 mM MgCl_2_, 1 mM DTT, 80 mM KCl, hybrid RNA/DNA (5′-GTTTTCTTTTCCCCCCTGAC-3′-Fluorescein, 5′-CAAAAGAAAAGGGGGGACUG-3′-Dabcyl) and 2 nM RT. The reaction mixture was incubated for 1 h at 37°C; the reaction was stopped by addition of EDTA and products were measured with a Victor 3 (Perkin Elmer model 1420-051) equipped with filters for 490/528 nm (excitation/emission wavelength).

### HIV-1 IN/LEDGF HTRF LEDGF Dependent Assay

Recombinant IN and LEDGF/p75 were purified as described (Esposito et al., 2015). The IN-LEDGF/p75 dependent assay allows to measure the inhibition of 3′ processing and strand transfer IN reactions in presence of recombinant LEDGF/p75 protein, as previously described (Tintori et al., 2015). Briefly, 50 nM IN was pre-incubated with increasing concentration of compounds for 1 h at room temperature in reaction buffer containing 20 mM HEPES pH 7.5, 1 mM DTT, 1% glycerol, 20 mM MgCl_2_, 0.05% Brij-35 and 0.1 mg/ml BSA. DNA donor substrate, DNA acceptor substrate and 50 nM LEDGF/p75 protein were added and incubated at 37°C for 90 min. After the incubation, 4 nM of Europium-Streptavidin were added to the reaction mixture and the HTRF signal was recorded using a Perkin Elmer Victor 3 plate reader using a 314 nm for excitation wavelength and 668 and 620 nm for the wavelength of the acceptor and the donor substrates emission, respectively.

### Cell-based HIV Assay

The procedure to determine anti-HIV activity and cytotoxicity of the compounds in human lymphocyte MT-4 cells was published elsewhere (Pannecouque et al., 2008). Briefly, serial dilutions of the compounds were added to 96-well plates containing the MT-4 cells. To the virus-infected wells, 100–300 CCID_50_ (50% cell culture infectious dose-50%) of HIV-1 (strain IIIB) or HIV-2 (strain ROD) was added. The mock-infected wells received the compounds without the virus. After 5 days incubation, the spectrophotometric MTT assay was performed to determine the effect of the compounds on the viability of the mock- and HIV-infected cells. The CC_50_ was defined as the compound concentration that reduced the viability of the mock-infected MT-4 cells by 50%. The concentration achieving 50% protection from the virus-induced cytopathic effect was defined as the 50% effective concentration (EC_50_).

### Molecular Modeling

Crystal structures of the full-length mutant HIV-1 RT containing RNase H domain and the PFV intasome as HIV-1 IN model, were retrieved from RCSB Protein Data Bank (accession codes 3LP2 and 5FRM, for RNase and IN, respectively). While the 5FRM was used as it is, the 3LP2 crystal was optimized by retro-mutation to the wild type by substitution at 103 position of Asparagine with Lysine, and then by insertion of the missed residue Arg557, as previously described (Budihas et al., 2005). Next, for both proteins, solvent and ligand molecules were removed, hydrogens were added, and partial atomic charges were assigned according to Amber99 force field, using MOE platform (Molecular Operating Environment [MOE], 2009).

Ligands were constructed using the builder feature implemented in MOE, and their energy was minimized until a convergence gradient of 0.01 kJ (mol Å)^-1^ was reached using the MMFF94x force field. Then, ligands were docked into the catalytic pocket of both protein models using Alpha Triangle Placement method. All docking parameters were kept as default. The best docking pose of each ligand has been considered for discussion and graphical representation.

## Results

### Compounds Tested

*N′*-acylhydrazones **1–23** (**Figure 1**), prepared in high yields as previously described (Congiu and Onnis, 2013; Carcelli et al., 2016; Kim et al., 2016), were fully characterized by spectroscopic tools, mass spectrometry and elemental analysis (see Supplementary Materials). To modulate the lipophilicity and hydrogen-bonding capabilities, we considered the *N′*-(2,3-dihydroxybenzylidene)-scaffold and the *N′*-(2-hydroxy-3-methoxybenzylidene)-analog and modified the acylhydrazonic substituent by introducing the heptyl (**1** and **2**), phenyl (**3** and **4**), 2-hydroxyphenyl (**5** and **6**) and 3,4,5-trihydroxyphenyl moiety (**14** and **15**). In order to investigate the role of hydroxyl substituents, compounds carrying none (**11**), one (**13**), two (**14**–**18**) or three (**20**–**23**) hydroxyl groups on the aromatic ring A and a 3,4,5-trihydroxy moiety in B were synthesized.

### Effect of Compounds on HIV-1 Functions

**FIGURE 2:**
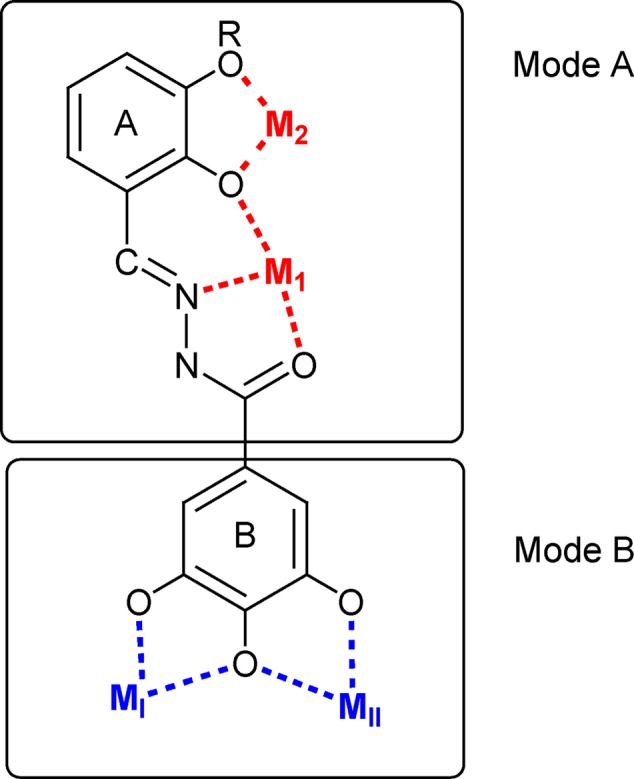
**Schematic representation of the possible binding modes of the studied *N′*-acylhydrazones**.

*N′*-acylhydrazones **1–23** were tested for their ability to inhibit HIV-1 RT-associated RNase H activity as well as HIV-1 IN activity in the presence of the human LEDGF/p75 cofactor (Cherepanov et al., 2005) (**Table 1**). Moderate potency of RNase H inhibition (IC_50_ about 60 μM) was observed when *N′*-2,3-dihydroxy benzylidene heptyl hydrazone **1** and its analog **2** (**Figure 1**) were assayed. This potency of inhibition was completely abolished when the heptyl chain was replaced by an aromatic group (compounds **3** and **4**). No inhibition of the RNase H was observed by the 2,3-dihydroxybenzylidene ligand **5**, while the 2-hydroxy-3-methoxy analog **6** had moderate activity (IC_50_ = 37 μM). *N′*-acylhydrazones **1–6** did not inhibit IN activity in the presence of the LEDGF cofactor. We then investigated the influence of hydroxyl substitution on the aromatic ring A (**Figure 2**) within the series **7–10**, which possesses three hydroxyls at varying positions in A. In this series, the position of the hydroxyl substituent improved the potency of inhibition against RNase H function. In fact, compound **7** inhibits the RNase H activity with an IC_50_ value of 25 μM, resulting around sixfold less potent than compound **10**, and revealing the crucial role of the hydroxyl substituent in engaging efficient interactions with the protein active site and in modulating the activity of the chelating inhibitor. Then, we investigated the role of different substitutions of A on *N′*-acylhydrazones carrying the gallic moiety as B (**Figure 2**): series **11–18** showed interesting potency of inhibition against the RNase H function (IC_50_ values ranging from 0.9 to 3.2 μM), while only compounds **16** and **18** inhibited the IN reaction (IC_50_ = 69 and 16 μM, respectively).

**Table 1:** Inhibitory effect of compounds **1–23** on HIV-1 RT-associated RNase H function and HIV-1 IN activity.

Compound	HIV-1 RNase H^a^ IC_50_ (μM)	HIV-1 IN LEDGF-dependent integration^b^ IC_50_ (μM)
(1)	61 ± 11	>100
(2)	62 ± 2	–
(3)	>100	>100
(4)	>100	–
(5)	>100	–
(6)	37 ± 5	>100
(7)	25.3 ± 7.6	7.1 ± 0.2
(8)	8.8 ± 3.1	30 ± 1
(9)	9.6 ± 3.4	9.4 ± 0.4
(10)	4.1 ± 0.2	>100
(11)	2.6 ± 0.6	>100
(12)	3.2 ± 0.5	>100
(13)	2.3 ± 0.7	>100
(14)	3.1 ± 0.5	>100
(15)	0.92 ± 0.02	>100
(16)	2.1 ± 0.1	69 ± 5
(17)	2.0 ± 0.5	>100
(18)	1.7 ± 0.1	16 ± 3
(19)	>100	>100
(20)	0.17 ± 0.002	0.085 ± 0.025
(21)	0.62 ± 0.10	0.08 ± 0.01
(22)	9.7 ± 1.6	1.8 ± 0.1
(23)	0.18 ± 0.03	0.165 ± 0.025
RDS1643	7.5 ± 0.9	ND
RAL	>50	0.058 ± 0.01

*^a^Compound concentration required to reduce the HIV-1 RT-associated RNase H activity by 50%. Mean values ± of three independent experiments. ^b^Compound concentration required to inhibit the HIV-1 IN catalytic activities by 50% in the presence of LEDGF. Mean values ± of three independent experiments.*

We then tested the series **20**–**23**, in which the *N′*-acylhydrazone scaffold carries three hydroxyl substituents on the aromatic ring A. When the OH groups were present in **2, 4** and **6** positions (compound **22**), a potency of inhibition in the micromolar range was observed for both viral enzymes (IC_50_ = 9.7 μM for RNase H and 1.8 μM for IN). When moving the 6-OH function to position **5**, for instance in compound **21**, the potency of inhibition was considerably improved, with IC_50_ values in the sub-micromolar range for HIV RNase H (IC_50_ = 0.62 μM) and in the nanomolar range for HIV IN (IC_50_ = 0.08 μM). Again, when a 3,4,5-trihydroxy substituent was present (compound **23**), the potency of RNase H inhibition was similar to the one of compound **21**, while inhibition of HIV IN was less efficient (IC_50_ = 0.16 μM). The optimal combination resulted in the compound **20**, which bears a 2,3,4-trihydroxybenzylidene moiety as A and showed inhibition in the nanomolar range for both HIV RNase H and IN (IC_50_ = 0.175 and 0.085 μM, respectively).

In cell-based HIV assays, the best result was obtained for compound **18** with an EC_50_ value for HIV-1 of 17 ± 4 μM (mean ± SD of two independent tests; data not shown). This molecule produced 55 to 82% inhibition of HIV-1-induced cytopathic effect at a concentration of 25 μM. Since its CC_50_ was 61 μM, its selectivity index was unfortunately too low to perform mechanistic studies. Besides, weak and poorly reproducible HIV-1 inhibition was seen for compounds **21–23** with EC_50_ values in the range of 2–20 μM and CC_50_ values ∼37 μM.

### Docking Studies

In order to explore the putative binding mode of the prototype compounds toward each target enzyme, a series of computational docking studies on some representative compounds was assessed. In addition to the most effective compound **18**, we also selected the compound **23**, starting from the observation that among the reported inhibitors within the **1–23** series, whose carrying a gallic moiety on the *N′*-acylhydrazone scaffold are endowed with the highest inhibitory activities against both enzymes. Additionally, to highlight some critical features that determine the dual inhibitory activity, compound **15** was chosen as a comparison, because it shows a good RNase H inhibitory activity (IC_50_ 0.92 μM), but it is devoid of anti-IN activity (IC_50_ > 100 μM).

**FIGURE 3:**
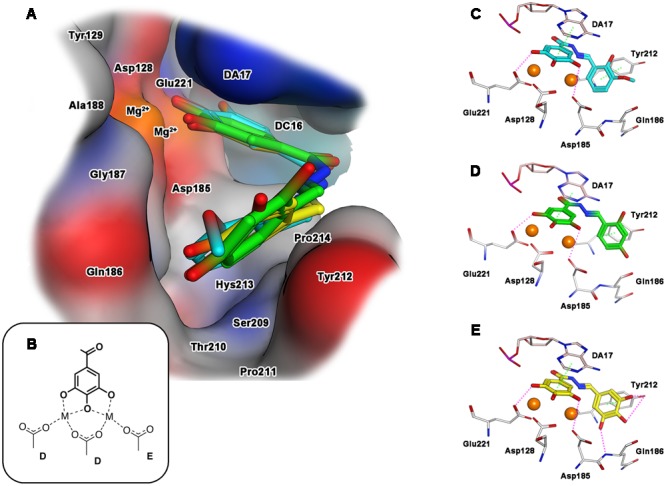
**Binding mode of the top-ranked docking results for compounds **15** (cyan), **18** (green) and **23** (yellow) in the HIV-1 IN catalytic site: (A)** superimposition of predicted conformations for ligands **15, 18**, and **23** within the catalytic pocket displayed as surface (blue, red, gray and orange colors indicate mildly polar, hydrophilic, hydrophobic and metal cofactors regions, respectively); **(B)** schematized chelating mode for the model ligands; **(C–E)** close view of predicted binding mode, where relevant residues interacting with ligands are depicted as thick lines. Metal cofactors are represented as orange spheres, hydrogen bonds (purple) and arene-arene or arene-cation (green) interactions are depicted as dashed lines.

Concerning the interaction with IN, when ligands **15, 18** and **23** were docked into the catalytic pocket of the PFV intasome (**Figure 3A**), all of them showed a common binding mode, involving the ring B: the gallic moiety is directed toward the metal ions, providing coordination in full accordance with the proposed mode B (**Figures 2, 3B**). In particular, the gallic moiety appeared to be located in close proximity to the catalytic triad, and this makes also possible the formation of two accessory hydrogen bonds between the carboxylate groups of Asp185 and Asp221, and the hydroxyl groups in positions **3** and **5** of the gallic ring B (**Figures 3C–E**). Moreover, rings A and B are engaged in arene–arene interactions with Tyr212, and with the aromatic scaffold of the adenine 17 of the viral nucleic acid (crystallized in the PFV intasome that we used as a model), respectively. Furthermore, for the docked compounds, additional contacts with residues Asp185, Gln186, Tyr212, and Pro214 were detected.

Analyzing the structure of compounds with the highest IN inhibition activity, such as compounds **18** and **23**, it can be observed that they are characterized by the presence of three hydroxyl substituents on aromatic ring A. These functionalities would effectively stabilize the ligand-target complex by means of two additional hydrogen bonds: the first with the hydroxyl group of Tyr212, and the second one with the amide NH of Gln186 (**Figure 3E** for **23**). Deletion of one hydroxyl group, as in compound **18**, significantly reduced the ability to inhibit IN, and only a residual inhibitory potency was detected. Also in the case of IN inactive compound **15**, the 2-hydroxy-3-methoxy motif did not show such interactions, probably due to the presence of only one hydrogen donor function, and/or to the steric hindrance caused by the methoxy group that prevents the correct orientation of the ring A within the cavity formed by Gln 186 and Tyr212. Overall, these information would corroborate the hypothesis that the inhibition of IN by these compounds is significantly influenced by the stabilization effect of the substituents present on the aromatic ring A.

Again, a close similarity between the hypothetical disposition of our compounds and the crystal structure of marketed IN inhibitor Raltegravir was observed (Supplementary Figure S1). In particular, the gallic moiety aligned with the 4-carboxamide-5-hydroxy-1-methyl-6-oxopyrimidine ring of Raltegravir, where the three hydroxyl functions of gallate overlap with the coordinating oxygens of raltegravir, while the ring A of **18** appears to mimic the oxadiazole ring.

**FIGURE 4:**
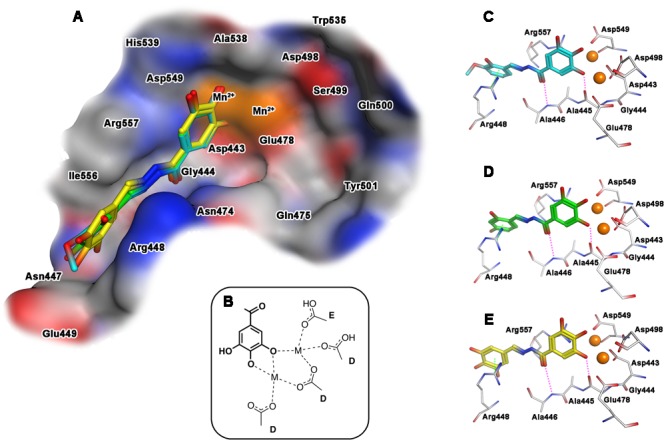
**Binding mode of the top-ranked docking results for compounds **15** (cyan), **18** (green) and **23** (yellow) at the HIV-1 RNase H catalytic site: (A)** superimposition of predicted conformations for ligands **15, 18**, and **23** within the catalytic pocket displayed as surface (blue, red, gray and orange colors indicate mildly polar, hydrophilic, hydrophobic and metal cofactors regions, respectively); **(B)** schematized chelating mode for the model ligands; **(C–E)** close view of predicted binding mode, where relevant residues interacting with ligands are depicted as thick lines. Metal cofactors are represented as orange spheres, hydrogen bonds (purple) and arene-arene or arene-cation (green) interactions are depicted as dashed lines.

When the docking of the interaction with the selected compounds and the RNase H domain was performed, results showed a slightly different behavior. In **Figure 4** the best docking poses obtained for compounds **15, 18** and **23** within the catalytic pocket of RNase H are represented. The predicted binding modes of the ligands involve also in this case the gallic moiety as chelating motif toward the metal ions, while the remaining portion of the molecules accommodates inside a narrow pocket lined by residues Gly444-Glu449, Arg557, and Asn474, and ring A stacked between Arg448 and Arg557 (**Figure 4A**). The main stabilizing interactions can be attributed to: (a) an arene–cation interaction with the aromatic ring A and the guanidinium group of Arg448; (b) a polar/hydrophilic interaction between ring A and the hydrazone group with Arg557; (c) a hydrogen bond with the carbonyl of hydrazone group and the amide NH of Ala446 and (d) a hydrogen bond between the hydroxyl group in position 3 of gallic moiety and Gly444 (**Figures 4C–E**). As observed for IN, also in this case the different inhibitory activities of the ligands seems related to the substituents on ring A.

## Discussion

The coordinating versatility of *N′*-acylhydrazone ligands is well-known (Albrecht et al., 2009; Ray et al., 2009; Rogolino et al., 2015). If a 2-hydroxy substituted phenyl ring is present on the backbone of the ligand, it can coordinate one or, depending on denticity, two metal centers (M_1_ and M_2_ in **Figure 2**). Moreover, if a gallic moiety is introduced as B (**Figure 2**), an additional coordinating mode arises (M_I_ and M_II_). In our recent studies, this versatility was demonstrated to be at the basis of efficient inhibition of influenza virus PA endonuclease, due to chelation of the two metal ions in the catalytic center of the enzyme (Carcelli et al., 2016). Hence we wanted to verify whether this versatility could be exploited for the development of effective dual chelating inhibitors of other metal-dependent enzymes, like HIV-1 RNase H and IN, with two Mg(II) ions in the catalytic site.

In fact, while most of the tested compounds were able to inhibit the RNase H function, compounds **7–9** showed also to be able to inhibit the HIV-1 IN, envisaging the possibility to design dual inhibitors, with a synergic effect against both HIV RNase H and IN. Of note, the activity of compound **10** toward RNase H is particularly interesting. In fact, compound **10**, In fact, since **10** does not carry the 2-hydroxyphenyl group in A, it lacks the possibility to chelate in a tridentate ONO fashion (Mode A in **Figure 2**); however, it can coordinate two cations by means of its three OH groups. This chelating mode was indeed observed in the crystal structure of **23** complexed with the N-terminal part of the influenza PA endonuclease, which showed that **23** chelates the two manganese (II) cofactors through its gallic moiety (Carcelli et al., 2016).

Results on compounds **10**–**23** indicated that more stringent requirements should be met to obtain efficient inhibition of HIV IN with these ligands, when none (**11**), one (**13**) or two (**14**–**18**) hydroxyl groups are present on aromatic ring A, or even when this is replaced by a pyridine moiety (**12**). Compounds **11**–**17** proved to be selective RNase H inhibitors. The goal of identifying dual chelating inhibitors of both HIV-1 RNase H and IN, was fulfilled in compound **18** and in the series **20**–**23**, where the *N′*-acylhydrazone scaffold carries three hydroxyl substituents on the aromatic ring A. In particular, compound **18** inhibits RNase H function ninefold more potently that IN activity and the HIV replication in the same micromolar range. Compounds **20–23** inhibit both HIV enzymes more potently than compound **18** and their inhibitory ability strongly depends on the position of the hydroxyl substituents, but a different trend has been detected for each enzyme, presumably attributable to the interactions established within the two different active sites. Compounds **20–23**, unfortunately, showed poor reproducibility in HIV replication assay, compared to compounds **18**. This can be due to lower solubility in cell culture, which prevented from obtaining reliable data for this series. Compounds **18**, on the contrary, with inhibition of HIV replication of 17 μM, can be considered a better hit. For compounds **11**–**18**, chelation of the metal cofactors according to mode B (**Figure 2**) could be hypothesized. This is consistent with (i) the activity of compound **11** that cannot exhibit the chelating mode A because it lacks the 2-OH group; (ii) the inactivity of compound **19**, where the gallic moiety is replaced by a 3,4,5-trimethoxy benzene ring. Evidently, the 3,4,5-trihydroxybenzyl moiety in B is essential, but not sufficient to ensure potent enzyme inhibition, since the interactions of ring A, for example with the amino acid side chains of the protein, appear to be crucial in modulating the inhibitory activity.

For the compounds **20–23**, we hypothesize that the inhibitory activity of this gallic moiety-containing compound series is determined by (i) the ability of chelating the metal ions in the active site of the enzyme; and (ii) the presence and position of the hydroxyl substituents in A, which may possibly yield relevant ligand–protein interactions (e.g., through hydrogen bonds).

It has been reported that hydrazones have an interesting dual inhibition profile, since they inhibit both RT-associated RNase H and RDDP functions (Borkow et al., 1997). In particular, hydrazones were reported to inhibit the RNase H function (i) binding to an allosteric site located between the polymerase active site and the non-nucleoside reverse transcriptase inhibitor (NNRTI) pocket (Himmel et al., 2006); (ii) binding to a site located between the RNase H active site and the connection domain (Gong et al., 2011). Differently, our data support the hypothesis that the present metal-chelating *N′*-acylhydrazone series bind as chelating agents into the RNase H active site.

Intriguingly, striking similarities can be seen in the coordination modes proposed for the best compound **18** and compounds **15** and **23** compared to those of some 3-hydroxypyrimidine-2,4-dione-5-N-benzylcarboxamides, recently described as potent inhibitors of HIV-1 IN and RNase H (Wu et al., 2016). In fact, compounds **15, 18** and **23** are predicted to establish secondary interactions with PFV residues Asp185, Tyr212, and Glu221, which correspond to HIV IN Asp116, Tyr143 and Glu152 (Dayer, 2016). Moreover, compound **23** establish additional secondary interactions with the hydroxyl group of Tyr212 and with the nitrogen of the peptidic bridge between Asp185 and Gln186. Importantly, while residue Tyr143 is known to be involved in the interaction with the first generation IN Inhibitors (Corona et al., 2016), both the catalytic Asp116 and Asn117 residues are part of a short, highly conserved region (H114-G118) (Ceccherini-Silberstein et al., 2009), that bridges the β4 sheet, with the α5 helix. Also of note the fact that among the residues hypothesized to be involved in the interaction between *N′*-acylhydrazone derivatives and the HIV-1 RT RNase H domain, some exhibit a high degree of conservation, even among patients treated with RT inhibitors (Santos et al., 2008). In particular, residue Asn474 is part of the RNase H primer grip motif and its mutation has been shown to determine to a strong decrease in viral fitness (Dash et al., 2008). Interestingly, residue Asn474 has been previously shown to play a crucial role also for pyrrolyl diketoacids binding (Corona et al., 2014a) and hence it seems to be involved in the binding of chelating agents to the RNase H active site.

Finally, aiming to develop dual HIV-1 RNase H – IN inhibitors, it is also worth noting that the predicted interactions obtained for the model ligands toward IN and RNase H would suggest two different coordinating modes: while for IN it can reasonably proposed the binding mode B (**Figure 3B**), in the case of RNase H an alternative coordinative hypothesis can be invoked (**Figure 4B**). In fact, docking results for ligands **15, 18** and **23** within the RNase H active site reveal that the gallic moiety is oriented perpendicularly to the plane formed by metal cofactors and the coordinating residues, with only two hydroxyl groups involved in the chelation (**Figure 4B**). This unusual orientation could depend on the presence, in the RNase H active site, of a fourth residue (i.e., Asp549), which is able to provide for an additional coordinating bond with a metal ion, thus blocking a coordination site of the metal. This essential information is clearly to be taken into account for further drug development of dual target inhibitors.

## Conclusion

The metal-chelating *N′*-acylhydrazones derivatives are a good source to design dual inhibitors targeted to both HIV-1 RT-associated RNase H and IN activities. The best identified analog, compound **18**, inhibits in the micromolar range both RNase H and IN functions and is also able to inhibit viral replication in the cell-based antiviral assay. The computational docking studies performed support the hypothesis that the *N′*-acylhydrazones derivatives can chelate the metal ions in both HIV-1 RNase H and IN active sites, and that the functional determinants for their efficacy are different with respect to the two enzymes. This information, together with the one that the binding of these compounds in the enzyme catalytic sites can be stabilized by the interaction with conserved amino acid residues, will be essential for the development of further analog optimization.

## Author Contributions

All authors have given approval to the final version of the manuscript. MC, DR, and AG: drug design and chemical synthesis. NP: docking studies. FE, AnC, AlC, CP, LN, and ET: biological studies. MC, DR, ET, and FE wrote the paper.

## Conflict of Interest Statement

The authors declare that the research was conducted in the absence of any commercial or financial relationships that could be construed as a potential conflict of interest. The reviewer SH and handling Editor declared their shared affiliation and the handling Editor states that the process nevertheless met the standards of a fair and objective review.

## Abbreviations

CC_50_: 50% cytotoxic concentration
DTT: dithiothreitol
EC_50_: half maximal effective concentration
HIV-1: Human Immunodeficiency Virus type 1
HTRF: Homogeneous Time Resolved Fluorescence
IC_50_: half maximal inhibitory concentration
IN: integrase
LEDGF: lens epithelium-derived growth factor
PFV: prototype foamy virus
RNase H: ribonuclease H
RT: reverse transcriptase
