# Supplementary material for: Chelation Motifs Affecting Metal-dependent Viral Enzymes: N′-acylhydrazone Ligands as Dual Target Inhibitors of HIV-1 Integrase and Reverse Transcriptase Ribonuclease H Domain
Source: Front Microbiol. 2017 Mar 20;8:440. doi: 10.3389/fmicb.2017.00440 (PMC5357622; doi:10.3389/fmicb.2017.00440)
Supplement: Supplementary file 1 [file Data_Sheet_1.pdf]

Supporting Information

Chelation motifs affecting metal-dependent viral enzymes: *N'*-acylhydrazone ligands as dual target inhibitors of HIV-1 Integrase and Reverse Transcriptase Ribonuclease H Domain

*Mauro Carcelli[1], Dominga Rogolino[1,2], Anna Gatti[1,2], Nicolino Pala[3], Angela Corona[4], Alessia Caredda[4], Enzo Tramontano[4,5], Christophe Pannecouque[6], Lieve Naesens[6], Francesca Esposito[4]**

[1]Department of Chemistry, University of Parma, Parco Area delle Scienze 17/A, I-43124 Parma, Italy;[2]CIRCMSB (Research Interuniversity Consortium Chemistry of Metals in Biological Systems) Parma Unit, University of Parma, Italy; [3]Department of Chemistry and Pharmacy, University of Sassari, Via Vienna 2, 07100 Sassari, Italy; [4]Department of Life and Environmental Sciences, University of Cagliari , Cittadella Universitaria SS554, 09042 Monserrato (CA), Italy; [5]Genetics and Biomedical Research institute, National Research Council (CNR), Monserrato(CA), Italy; [6]Rega Institute for Medical Research, KU Leuven, B-3000 Leuven, Belgium.

***Corresponding author.**

Francesca Esposito, tel. +39 070/6754533; e-mail address francescaesposito@unica.it

Synthesis of compound 1-23.

3,4,5-trihydroxybenzohydrazide and 3,4,5-trimethoxybenzohydrazide were obtained by reaction of the corresponding methyl esters with hydrazine monohydrate. Hydrazine was added to an ethanol suspension of the ester and stirred at room temperature until the solute completely dissolved. After this time, the reaction mixture was refluxed overnight. On concentrating the solution, a precipitate was observed, which was filtered and washed with cold ethanol.

To a solution of the aldehyde in absolute ethanol or toluene, an equimolar amount of the proper hydrazide dissolved in the same solvent was added. The mixture was refluxed for 6 hours, cooled at room temperature and concentrated in vacuum. The resulting precipitate was filtered off, washed with cold ethanol and dried in vacuum.

3,4,5-trihydroxybenzohydrazide. Light brown powder. Yield: 70%. ^{1}H-NMR (DMSO-d_6, 25°C), δ: 4.36 (s, br, 2H, NH_2), 6.79 (s, 2H, H_{arom}), 9.24 (s, br, 1H, NH); IR (cm^{-1}): ν_{NH} = 3424, 3390, 3296; $\nu_{C=O}$ = 1600.

3,4,5-trimethoxybenzohydrazide. White powder. Yield: 63%. ^{1}H-NMR (DMSO-d_6, 25°C), δ: 3.69 (s, 3H, OCH_3), 3.81 (s, 6H, OCH_3), 4.47 (s, br, 2H, NH_2), 7.16 (s, 2H, H_{arom}), 9.72 (s, br, 1H, NH); IR (cm^{-1}): ν_{NH} = 3455; $\nu_{C=O}$ = 1697; $\nu_{C=N}$ = 1596.

***N'*-(2,3-dihydroxybenzylidene)heptylhydrazide (1).**Yield = 95%.^{1}H-NMR (DMSO-d_6, 25°C), δ: 0.83-0.86 (m, CH_3); 1.16-1.26 (m, CH_2); 1.54-1.59 (m, CH_2); 2.17-2.29 (m, CH_2); 2.53-2.59 (m, 6.64-6.73), (m, overlapping isomers, ArH), 6.81-6.84 (m, overlapping isomers, ArH), 6.92 (d, isomer E, ArH), 7.03 (d, isomer Z, ArH), 8.23 (s, HC=N, isomer E), 8.28 (s, HC=N, isomer Z), 9.21 (s, br, OH), 9.49 (s, br, OH), 11.06, 11.20, 11.59 (s, br, NH+OH). MS(EI, 70 eV) m/z (%) = 301 ([M+Na]$^+$, 100);IR (cm^{-1}): ν_{NH} = 3490; ν_{OH} = 2922-2941; $\nu_{C=O}$ = 1663. Anal. Calcd for $C_{15}H_{22}N_2O_3 \cdot 1/2H_2O$: C 62.70; H 8.07; N9.75. Found: C 62.84; H 7.97; N 9.88.

***N'*-(2-hydroxy-3-methoxybenzylidene)heptylhydrazide (2).** Yield = 41%. ^{1}H-NMR (DMSO-d_6, 25°C), δ (overlapping isomers): 0.83-0.86 (m, CH_3); 1.26-1.27 (m, CH_2); 1.55-1.59 (m, CH_2); 2.18-2.23 (m, CH_2); 2.52-2.57 (m, CH_2), 3.79 (s, br, OCH_3), 6.80-6.86 (m, ArH), 6.94-7.01 (m, ArH),

7.08 (d, isomer E, ArH), 7.20 (d, isomer Z, ArH), 8.27 (s, HC=N, isomer E), 8.34 (s, HC=N, isomer Z), 9.54 (s, br, OH), 10.96, 11.25, 11.57 (s, br, NH+OH). MS(EI, 70 eV) m/z (%)= 292.2 ($[M]^+$, 24);IR (cm^{-1}): ν_{NH} = 3182; ν_{OH} = 3072, 2917; $\nu_{C=O}$ = 1663. Anal. Calcd for $C_{16}H_{24}N_2O_3$: C 65.73; H 8.27; N9.58. Found: C 65.76; H 8.42; N9.45.

***N'*-(2,3-dihydroxybenzylidene)benzoylhydrazide (3).** Yield = 32%. ^{1}H-NMR (DMSO-d_6, 25°C), δ:6.74 (t, J = 7.8 Hz, 1H, ArH),6.86 (d, J = 7.2 Hz, 1H, ArH), 6.97 (d, J = 7.2 Hz, 1H, ArH), 7.52-7.61 (m, 3H; ArH), 7.94 (d, 2H; J = 7.2, ArH), 8.59 (s, 1H; HC=N), 9.28 (s, br, 1H; OH), 11.16 (s, br, 1H; NH), 12.14 (s, br, 1H; OH). MS(EI, 70 eV) m/z (%) = 256.2 ($[M]^+$, 100);IR (cm^{-1}): ν_{NH} = 3280; ν_{OH} = 3047; $\nu_{C=O}$ = 1658;$\nu_{C=N}$ = 1527. Anal. Calcd for $C_{14}H_{12}N_2O_3·1/4H_2O$: C 64.48; H 4.83; N10.74. Found: C 64.64; H 4.71; N10.71.

***N'*-(2-hydroxy-3-methoxybenzylidene)benzoylhydrazide (4).** Yield = 89%. ^{1}H-NMR (DMSO-d_6, 25°C), δ:3.81 (s, 3H, OCH_3), 6.87 (t, J = 7.8 Hz, 1H, ArH),7.04 (d, J = 7.2 Hz, 1H, ArH), 7.15 (d, J = 7.2 Hz, 1H, ArH), 7.52-7.64 (m, 3H; ArH), 7.94 (d, 2H; J = 6.9, ArH), 8.65 (s, 1H; HC=N), 11.01 (s, br, 1H; NH), 12.05 (s, br, 1H; OH). MS(EI, 70 eV) m/z (%) =270.1 ($[M]^+$, 100); IR (cm^{-1}): ν_{NH+OH} = 2830-3072 (br); $\nu_{C=O}$ = 1657; ν_{OCH3} = 1249, 1076. Anal. Calcd for $C_{15}H_{14}N_2O_3·H_2O$: C 62.49; H 5.59; N9.72. Found: C 62.88; H 5.59; N9.94.

***N'*-(2,3-dihydroxybenzylidene)-2-hydroxybenzoylhydrazide (5).** Yield = 30%. ^{1}H-NMR (DMSO-d_6, 25°C), δ:6.75 (t, J = 7.8 Hz, 1H, ArH),6.87 (d, J = 7.2 Hz, 1H, ArH), 6.94-7.00 (m,3H, ArH), 7.43 (t, J = 7.8 Hz, 1H; ArH), 7.88 (d, 2H; J = 7.2, ArH), 8.64 (s, 1H; HC=N), 9.32 (s, br, 1H; OH), 11.03 (s, br, 1H; NH), 12.05 (s, br, 2H; OH). MS(EI, 70 eV) m/z (%) = 272.1 ($[M]^+$, 100); IR (cm^{-1}): ν_{NH+OH} = 2980-3256 (br); $\nu_{C=O}$ = 1635. Anal. Calcd for $C_{14}H_{12}N_2O_4$.: C 61.76; H 4.44; N10.29. Found: C 61.97; H 4.43; N10.20.

***N'*-(2-hydroxy-3-methoxybenzylidene)-2-hydroxybenzoylhydrazide (6).**Yield = 89%. ^{1}H-NMR (DMSO-d_6, 25°C), δ:3.82 (s, 3H, OCH_3),6.85-7.06 (m, 4H, ArH),7.17 (d, J = 7.5 Hz, 1H, ArH), 7.45 (t, J = 7.6 Hz, 1H; ArH), 7.89 (d, 2H; J = 7.5, ArH), 8.69 (s, 1H; HC=N), 10.87 (s, br, 1H; NH), 11.99 (s, br, 2H; OH). MS(EI, 70 eV) m/z (%)= 286.0 ($[M]^+$, 100); IR (cm^{-1}): ν_{NH+OH} = 2993-3211 (br); $\nu_{C=O}$ = 1606; $\nu_{C=N}$ = 1560; ν_{OCH3} = 1256, 1079. Anal.Calcd for $C_{15}H_{14}N_2O_4·1/2H_2O$: C 61.01; H 5.12; N9.49. Found: C 61.20; H 4.89; N9.58.

***N'*-(2,3,4-trihydroxyphenyl)-2-hydroxybenzoylhydrazide (7).** Yield = 75%. ^{1}H-NMR (DMSO-d_6, 25°C), δ: 6.41 (d, J = 6.4 Hz, 1H, ArH), 6.83 (d, J = 6.8 Hz, 1H, ArH), 6.95-7.00 (m, 2H, ArH), 7.46 (t, J= 7.4, 1H, ArH), 7.89 (d, J= 7.8, 1H, ArH), 8.52-8.54 (overlapping singlets, 2H; HC=N+OH), 9.54 (s, br, 1H; OH), 11.39 (s, br, 1H; NH), 11.91 (s, br, 2H; OH). MS(EI, 70 eV), m/z (%) =287.9 ($[M]^+$, 40); IR (cm^{-1}): ν_{NH} = 3292 (br); ν_{OH} = 3226 (br); $\nu_{C=O}$ = 1632. Anal. Calcd for $C_{14}H_{12}N_2O_5$: C 58.33; H 4.20; N 9.72. Found: C 58.45; H 4.12; N 9.96.

***N'*-(2,4,5-trihydroxyphenyl)-2-hydroxybenzoylhydrazide (8).** Yield = 84%. ^{1}H-NMR (DMSO-d_6, 25°C), δ: 6.35 (s, 1H, ArH), 6.93-6.98 (m, 3H, ArH), 7.44 (t, J = 6.9, 1H, ArH), 7.89 (d, J = 7.0, 1H, ArH), 8.50 (s, 1H; HC=N), 8.62 (s, br, 1H; OH), 9.61 (s, br, 1H; OH), 10.50 (s, br, 1H, NH), 11.81, 12.00 (s, br, 1H; OH). MS(EI, 70 eV), m/z (%) =288.1 ($[M]^+$, 20); IR (cm^{-1}): ν_{NH+OH} = 3274-3420 (br); $\nu_{C=O}$ = 1634. Anal. Calcd for $C_{14}H_{12}N_2O_5·H_2O$: C 54.90; H 4.61; N 9.15. Found: C 54.65; H 4.72; N 9.29.

***N'*-(2,4,6-trihydroxyphenyl)-2-hydroxybenzoylhydrazide (9).** Yield = 65%. ^{1}H-NMR (DMSO-d_6, 25°C), δ: 5.86 (s, 2H, ArH), 6.93-6.98 (m, 2H, ArH), 7.45 (t, J = 6.9, 1H, ArH), 7.88 (d, J = 7.0, 1H, ArH), 8.83 (s, 1H; HC=N), 9.89 (s, br, 1H; OH), 11.09 (s, br, 1H; NH), 11.95, 11.97 (s, br, 1H; OH). MS(EI, 70 eV), m/z (%) =288.1 ($[M]^+$, 20); IR (cm^{-1}): ν_{NH+OH} = 3100-3360 (br); $\nu_{C=O}$ = 1631. Anal. Calcd for $C_{14}H_{12}N_2O_5·H_2O$: C 54.90; H 4.61; N 9.15. Found: C 54.82; H 4.52; N 9.42.

***N'*-(3,4,5-trihydroxyphenyl)-2-hydroxybenzoylhydrazide (10).** Yield = 76 %. ^{1}H-NMR (DMSO-d_6, 25°C), δ: 6.72 (s, 2H, ArH), 6.92-6.97 (m, 2H, ArH), 7.43 (t, J = 7.1, 1H, ArH), 7.88 (d, J = 7.0, 1H, ArH), 8.19 (s, 1H; HC=N), 8.67 (s, br, 1H; OH), 9.18 (s, br, 2H; OH), 11.67 (s, br, 2H; NH), 12.03 (s, br, 1H; OH). MS(EI, 70 eV), m/z (%) =287.9 ($[M]^+$, 35); IR (cm^{-1}): ν_{NH+OH} = 3280-3321 (br); $\nu_{C=O}$ = 1637. Anal. Calcd for $C_{14}H_{12}N_2O_5$: C 58.33; H 4.20; N 9.72. Found: C 58.19; H 4.03; N 9.54.

***N'*-phenyl-3,4,5-trihydroxybenzoylhydrazide (11).** Yield = 70%. ^{1}H-NMR (DMSO-d_6, 25°C), δ: 6.93 (s, 2H; ArH), 7.44 (m, 3H, ArH), 7.68 (d, J = 7.9 Hz, 2H, ArH), 8.41 (s, 1H; HC=N), 8.84 (s, br, 1H; OH), 9.15 (s, br, 2H; OH), 11.54 (s, br, 1H; NH). MS(EI, 70 eV), m/z (%) = 272.1 ($[M]^+$, 20); IR (cm^{-1}): ν_{OH}= 3534,ν_{NH+OH} = 3226-3327, (br); $\nu_{C=O}$ = 1590. Anal. Calcd for $C_{14}H_{12}N_2O_4$.: C 61.76; H 4.44; N 10.29. Found: C 61.55; H 4.67; N 10.04.

***N'*-(2-pyridyl)-3,4,5-trihydroxybenzoylhydrazide (12).**Yield = 55%. ^{1}H-NMR (DMSO-d_6, 25°C), δ: 6.97 (s, 2H; ArH), 7.67 (m, 1H, ArH), 8.12-8.22 (m, 2H, ArH), 8.58 (s, 1H; HC=N), 8.71 (d, 1H; ArH). MS(EI, 70 eV), m/z (%) = 273.1 ($[M]^+$, 20); IR (cm^{-1}): ν_{OH}= 3531,ν_{NH+OH} = 3216-3297, (br); $\nu_{C=O}$ = 1598. Anal. Calcd for $C_{13}H_{11}N_3O_4$.: C 57.14; H 4.06; N 15.38. Found: C 57.35; H 4.10; N 15.43

***N'*-(2-hydroxy-benzylidene)-3,4,5-trihydroxybenzoylhydrazone (13).** Yield = 79%. ^{1}H-NMR (DMSO-d_6, 25°C), δ: 6.90-6.95 (m, 4H; ArH), 7.29 (t, 1H, J = 7.9 Hz, ArH), 7.47 (d, J = 7.8 Hz, 2H, ArH), 8.58 (s, 1H; HC=N), 8.89 (s, 1H; OH), 9.20 (s, br, 2H; OH), 11.49 (s, 1H; OH), 11.84 (s, br, 1H; NH). MS(EI, 70 eV), m/z (%) = 288.1 ($[M]^+$, 25); IR (cm^{-1}): ν_{OH}= 3534,ν_{NH+OH} = 3226-3327, (br); $\nu_{C=O}$ = 1590. Anal. Calcd for $C_{14}H_{12}N_2O_5$.: C 58.33; H 4.20; N 9.72. Found: C 58.67; H 4.25; N 9.80.

***N'*-(2,3-dihydroxybenzylidene)-3,4,5-trihydroxybenzoylhydrazide (14).** Yield = 83%. ^{1}H-NMR (DMSO-d_6, 25°C), δ: 6.73 (t, J = 7.8 Hz, 1H, ArH), 6.84 (d, J = 7.2 Hz, 1H, ArH), 6.90 (d, 1H, ArH), 6.95 (s, 2H; ArH), 8.53 (s, 1H; HC=N), 8.89 (s, br, 1H; OH), 9.12 (s, br, 1H; OH), 9.21 (s, br, 2H; OH), 11.41 (s, br, 1H; NH), 11.83 (s, br, 1H; OH). MS(EI, 70 eV) m/z (%) = 304.0 ($[M]^+$, 100);): ν_{NH+OH} = 3255 (br); $\nu_{C=O}$ = 1654. Anal. Calcd for $C_{14}H_{12}N_2O_6$.: C 55.27; H 3.98; N 9.21. Found: C 55.04; H 4.12; N 9.15.

***N'*-(2-hydroxy-3-methoxybenzylidene)-3,4,5-trihydroxybenzoylhydrazide (15).** Yield = 61%. ^{1}H-NMR (DMSO-d_6, 25°C), δ: 3.81 (s, 3H, OCH_3), 6.85 (t, J = 7.9 Hz, 1H, ArH), 6.94 (s, 2H; ArH), 7.03 (d, J = 8.1 Hz, 1H, ArH), 7.08 (d, 1H, J = 7.9 Hz, ArH), 8.58 (s, 1H; HC=N), 8.90 (s, br, 1H; OH), 9.19 (s, br, 2H; OH), 11.25 (s, br, 1H; NH), 11.80 (s, br, 2H; OH). ^{1}H-NMR (MeOD-d_4, 25°C), δ: 3.91 (s, 3H, OCH_3), 6.91 (t, J = 7.9 Hz, 1H, ArH), 7.03-7.07 (m, 3H; ArH), 7.23 (d, J = 7.6 Hz, 1H, ArH), 8.57 (s, 1H; HC=N). ^{13}C-NMR (MeOD-d_4, 25°C), δ: 55.43; 107.11; 113.72; 118.87; 119.10; 121.39; 122.90; 137.54; 145.61; 147.48; 148.17; 148.54; 164.75. MS(EI, 70 eV) m/z (%) = 318.0 ($[M]^+$, 100); IR (cm^{-1}): ν_{OH} = 3418; ν_{NH+OH} = 3222 (br); $\nu_{C=O}$ = 1664; $\nu_{C=N}$ = 1598; ν_{OCH3} = 1252, 1033. Anal. Calcd for $C_{15}H_{14}N_2O_6 \cdot H_2O$: C 53.57; H 4.80; N 8.33. Found: C 53.50; H 4.88; N 7.92.

***N'*-(2,5-dihydroxybenzylidene)-3,4,5-trihydroxybenzoylhydrazide (16).** Yield = 59%. ^{1}H-NMR (DMSO-d_6, 25°C), δ: 6.74 (m, 2H; ArH), 6.89 (s, 1H, ArH), 6.94 (s, 2H, ArH), 8.50 (s, 1H; HC=N), 8.89, 9.00 (br, 2H; OH), 9.22 (s, br, 1H; OH), 10.60 (s, br, 1H; NH), 11.74 (s, br, 1H; OH). MS(EI, 70 eV), m/z (%) = 304.1 ($[M]^+$, 100); IR (cm^{-1}): ν_{OH} = 3403 (br); ν_{NH+OH} = 3215 (br); $\nu_{C=O}$ = 1592. Anal. Calcd for $C_{14}H_{12}N_2O_6 \cdot H_2O$: C 52.18; H 4.38; N 8.69. Found: C 52.44; H 4.44; N 8.58.

***N'*-(2-hydroxy-5-methoxybenzylidene)-3,4,5-trihydroxybenzoylhydrazide (17).** Yield = 92%. ^{1}H-NMR (DMSO-d_6, 25°C), δ: 3.73 (s, 3H, OCH_3), 6.84-6.91 (m, 2H, ArH), 6.94 (s, 1H, ArH), 7.06 (s, 1H, ArH), 8.56 (s, 1H; HC=N), 8.97 (br, 3H; OH), 10.89 (s, br, 1H; NH), 11.83 (s, br, 1H; OH). MS(EI, 70 eV), m/z (%) = 318.3 ($[M]^+$, 100); IR (cm^{-1}): ν_{OH}= 3496,ν_{NH+OH} = 3178 (br); $\nu_{C=O}$ = 1654. Anal. Calcd for $C_{15}H_{14}N_2O_6$: C 56.60; H 4.43; N 8.80. Found: C 56.89; H 4.32; N 8.96.

***N'*-(2,4-dihydroxybenzylidene)-3,4,5-trihydroxybenzoylhydrazide (18).** Yield = 43%. ^{1}H-NMR (DMSO-d_6, 25°C), δ: 6.31 (s, 1H; ArH), 6.36 (dd, J = 7.9 Hz, 1H, ArH), 6.92 (s, 2H, ArH), 7.24 (d, J = 7.8 Hz, 1H, ArH), 8.45 (s, 1H; HC=N), 9.18 (s, br, 2H; OH), 9.93 (s, br, 1H; OH), 11.63 (m, br, 2H; NH+OH). MS(EI, 70 eV), m/z (%) = 304.1 ($[M]^+$, 100; IR (cm^{-1}): ν_{OH} = 3552; ν_{NH+OH} = 3261-3320 (br); $\nu_{C=O}$ = 1630; $\nu_{C=N}$ = 1565. Anal. Calcd for $C_{14}H_{12}N_2O_6$.: C 55.27; H 3.98; N 9.21. Found: C 55.02; H 3.88; N 9.20.

***N'*-(2,5-dihydroxybenzylidene)-3,4,5-trimethoxybenzoylhydrazone (19).** Yield = 90%. ^{1}H-NMR (DMSO-d_6, 25°C), δ: 3.74 (s, 3H, OCH3), 3.87 (s, 6H, OCH3), 6.74 (m, 2H, ArH), 7.00 (s, 1H, ArH), 7.27 (s, 2H, ArH), 8.59 (s, 1H; HC=N), 8.98 (br, 1H; OH), 10.34 (s, br, 1H; NH), 11.83 (s,

br, 1H; OH). MS(EI, 70 eV), m/z (%) = 346.3 ($[M]^+$, 100); IR (cm^{-1}): ν_{NH}= 3274,ν_{OH} = 3090-3160 (br); $\nu_{C=O}$ = 1654. Anal. Calcd for $C_{17}H_{18}N_2O_6$: C 58.96; H 5.24; N 8.09. Found: C 58.82; H 5.52; N 8.15.

***N'*-(2,3,4-trihydroxyphenyl)-3,4,5-trihydroxybenzoylhydrazide (20).** Yield = 25%. ^{1}H-NMR (DMSO-d_6, 25°C), δ: 6.38 (d, J = 9 Hz, 1H, ArH), 6.72 (d, J = 8.9 Hz, 1H, ArH), 6.92 (s, 2H, ArH), 8.41 (s, 1H; HC=N), 8.44 (s, br, 1H; OH), 8.86 (s, br, 1H; OH), 9.19 (s, br, 2H; OH), 9.40 (s, br, 1H; OH), 11.66 (s, br, 1H; NH), 11.72 (s, br, 1H; OH). MS(EI, 70 eV), m/z (%) = 319.9 ($[M]^+$, 40); IR (cm^{-1}): ν_{NH+OH} = 3298-3420 (br); $\nu_{C=O}$ = 1608. Anal. Calcd for $C_{14}H_{12}N_2O_7$.: C 52.51; H 3.78; N 8.75. Found: C 52.42; H 3.88; N 8.56.

***N'*-(2,4,5-trihydroxyphenyl)-3,4,5-trihydroxybenzoylhydrazide (21).** Yield = 83%. ^{1}H-NMR (DMSO-d_6, 25°C), δ: 6.32 (s, 1H, ArH), 6.79 (s, 1H, ArH), 6.91 (s, 2H, ArH), 8.38 (s, 1H; HC=N), 8.53 (s, br, 1H; OH), 8.83 (s, br, 1H; OH), 9.16(s, br, 2H; OH), 9.52 (s, br, 1H; OH), 10.83 (s, br, 1H, NH), 11.52 (s, br, 1H; OH). MS(EI, 70 eV), m/z (%) = 320.1 ($[M]^+$, 20); IR (cm^{-1}): ν_{NH+OH} = 3250-3300 (br); $\nu_{C=O}$ = 1635. Anal. Calcd for $C_{14}H_{12}N_2O_7$.: C 52.51; H 3.78; N 8.75. Found: C 52.35; H 4.02; N 8.67.

***N'*-(2,4,6-trihydroxyphenyl)-3,4,5-trihydroxybenzoylhydrazide (22).** Yield = 72%. ^{1}H-NMR (DMSO-d_6, 25°C), δ: 5.83 (s, 2H, ArH), 6.90 (s, 2H, ArH), 8.75 (s, 1H; HC=N), 8.85 (s, br, 1H; OH), 9.18 (s, br, 2H; OH), 9.77 (s, br, 1H; OH), 11.12 (s, br, 2H; NH+OH), 11.60 (s, br, 1H; OH). MS(EI, 70 eV), m/z (%) = 320.2 ($[M]^+$, 35); IR (cm^{-1}): ν_{NH+OH} = 3298-3420 (br); $\nu_{C=O}$ = 1608. Anal. Calcd for $C_{14}H_{12}N_2O_7$.: C 52.51; H 3.78; N 8.75. Found: C 52.74; H 3.51; N 8.53.

***N'*-(3,4,5-trihydroxyphenyl)-3,4,5-trihydroxybenzoylhydrazide (23).** Yield = 73%. ^{1}H-NMR (DMSO-d_6, 25°C), δ: 6.65 (s, 2H, ArH), 6.89 (s, 2H, ArH), 8.12 (s, 1H; HC=N), 8.55 (s, br, 1H; OH), 8.80 (s, br, 1H; OH), 9.11-9.14 (m, br, 4H; NH+OH), 11.26 (s, br, 1H; OH). MS(EI, 70 eV), m/z (%) = 320.2 ($[M]^+$, 35); IR (cm^{-1}): ν_{NH+OH} = 3244-3343 (br); $\nu_{C=O}$ = 1620. Anal. Calcd for $C_{14}H_{12}N_2O_7$.: C 52.51; H 3.78; N 8.75. Found: C 52.55; H 3.92; N 8.73.

Figure S1. Binding mode comparison: superimposition of the top conformation for **18** (green), selected as *N'*-acylhydrazone model compound, and the crystal structure of Raltegravir (yellow), into the IN PFV intasome active site. Target protein is represented by white cartoon and metal cofactors as orange spheres.
