# Supplementary figures and images for: Chelation Motifs Affecting Metal-dependent Viral Enzymes: N′-acylhydrazone Ligands as Dual Target Inhibitors of HIV-1 Integrase and Reverse Transcriptase Ribonuclease H Domain
Source: Front Microbiol. 2017 Mar 20;8:440. doi: 10.3389/fmicb.2017.00440 (PMC5357622; doi:10.3389/fmicb.2017.00440)

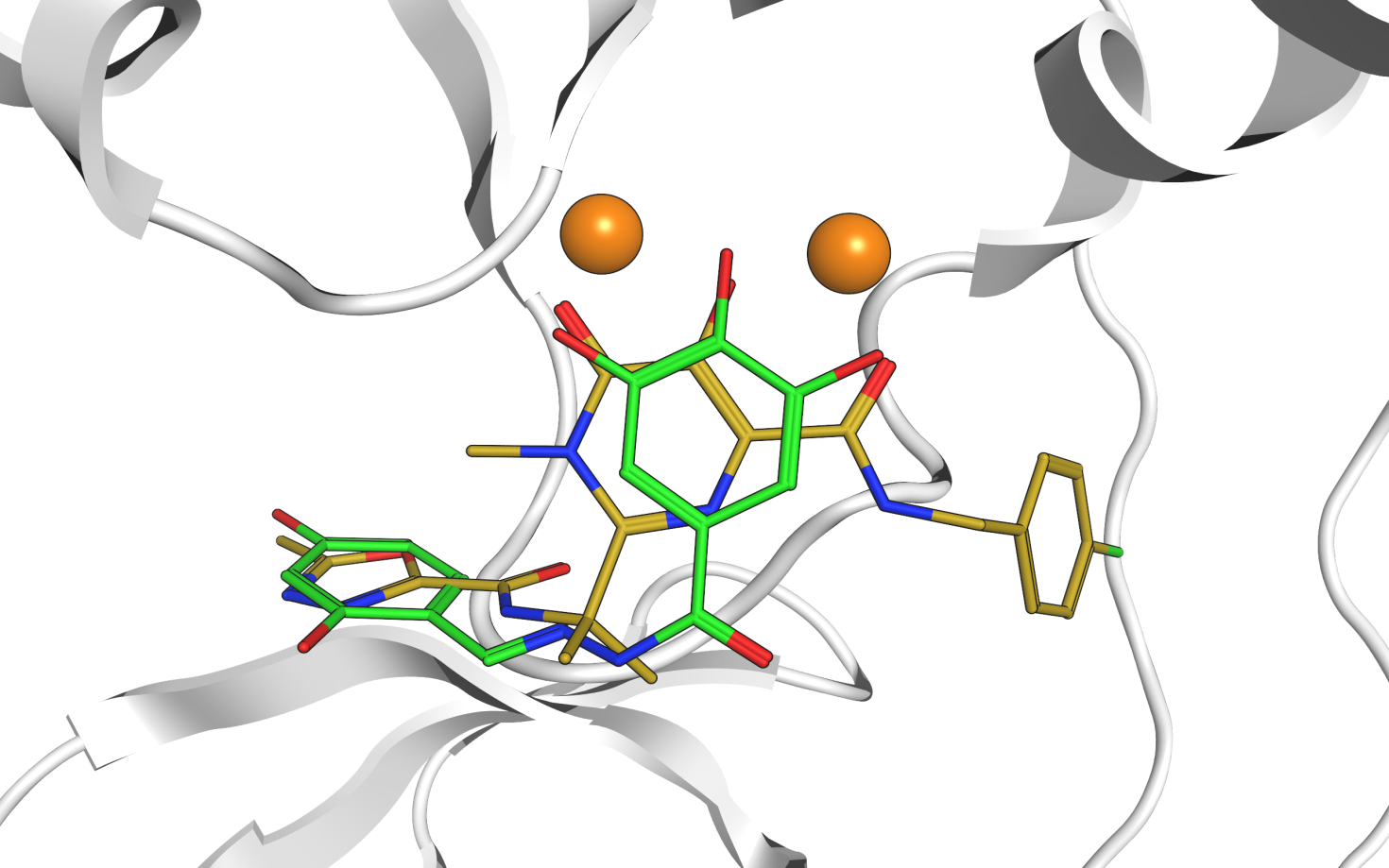

Supplement: Supplementary file 2 [file Image_1.TIF]
